# Membrane-Disrupting Activity of Cobra Cytotoxins Is Determined by Configuration of the N-Terminal Loop

**DOI:** 10.3390/toxins15010006

**Published:** 2022-12-20

**Authors:** Peter V. Dubovskii, Anastasia A. Ignatova, Anna S. Alekseeva, Vladislav G. Starkov, Ivan A. Boldyrev, Alexey V. Feofanov, Yuri N. Utkin

**Affiliations:** 1 Shemyakin-Ovchinnikov Institute of Bioorganic Chemistry, Russian Academy of Sciences, 117997 Moscow, Russia; 2 Bioengineering Department, Faculty of Biology, Lomonosov Moscow State University, 119234 Moscow, Russia

**Keywords:** cobra cytotoxin, antibacterial activity, cytotoxic activity, biological membrane, spatial structure, phospholipid liposomes, calcein leakage

## Abstract

In aqueous solutions, cobra cytotoxins (CTX), three-finger folded proteins, exhibit conformational equilibrium between conformers with either cis or trans peptide bonds in the N-terminal loop (loop-I). The equilibrium is shifted to the cis form in toxins with a pair of adjacent Pro residues in this loop. It is known that CTX with a single Pro residue in loop-I and a cis peptide bond do not interact with lipid membranes. Thus, if a cis peptide bond is present in loop-I, as in a Pro-Pro containing CTX, this should weaken its lipid interactions and likely cytotoxic activities. To test this, we have isolated seven CTX from *Naja naja* and *N. haje* cobra venoms. Antibacterial and cytotoxic activities of these CTX, as well as their capability to induce calcein leakage from phospholipid liposomes, were evaluated. We have found that CTX with a Pro-Pro peptide bond indeed exhibit attenuated membrane-perturbing activity in model membranes and lower cytotoxic/antibacterial activity compared to their counterparts with a single Pro residue in loop-I.

## 1. Introduction

Currently, there is a strong need for development of novel anticancer and antibacterial drugs [1], since both bacteria and cancer cells are able to acquire resistance to traditional drugs [2,3]. These new drugs are searched for, in particular, among toxins of animal origin [4,5,6]. A number of such toxins possess membrane activity [7,8,9], and this circumstance makes these molecules an attractive drug lead for new antibacterial/anticancer compounds. It is believed that if the plasma membrane of bacteria or cancer cells becomes the target of such compounds, then the development of their resistance should be significantly hindered [10,11,12].

**Figure 1:**
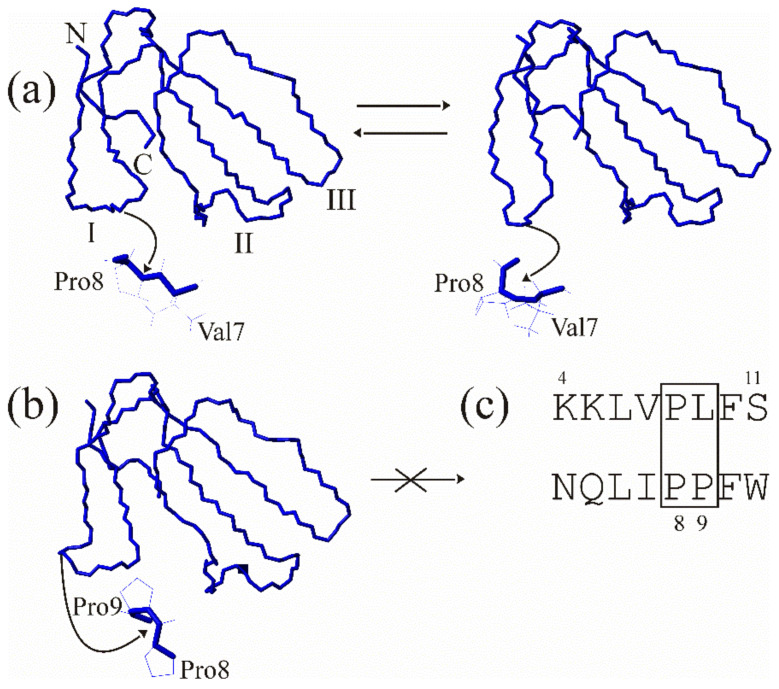
Conformational equilibrium of CTX in aqueous solution. (**a**) Major (left, pdb code 1CB9) and minor (right, 1CCQ) forms of cytotoxin 2 from *N. oxiana* in an aqueous solution. The major form is characterized by a trans-configuration of the Val7-Pro8 peptide bond while in the minor form this bond is in a cis-configuration (shown below). The N and C-termini are marked with N and C, respectively. The loops are numbered with Roman numerals. (**b**) For a toxin γ from *N. nigricollis*, only one form (pdb code 1TGX) with a cis-configuration between Pro8-Pro9 residues is present (this bond is shown below) (**c**) The equilibrium depends on the amino acid sequence within the tip of loop-I. The fragment of amino acid sequence of cytotoxin 2 from *N. oxiana* (upper sequence) is compared with that of toxin γ (lower sequence) from *N. nigricollis*. The substitution of the 9th residue to Pro (residues 8–9 are enclosed in the box) stabilizes the cis form. On all the panels only backbone atoms are shown. The orientation of all the molecules is identical. The side-chains in the inserts below the conformers (a, b) are shown in thin line, while backbone is in bold line. All inserts are magnified.

Membrane-active compounds, otherwise known as cytolytic polypeptides, are abundant in insect and snake venoms [13]. They are usually cationic and contain no or several disulfide bonds [14,15]. The former are known as linear polypeptides; the others belong to the class of disulfide-rich proteins. Cobra cytotoxins (or cardiotoxins, CTX) from the three-finger toxin (TFT) family, are examples of the latter toxins, isolated from either cobra or coral snake venom [16,17,18]. CTX are amphiphilic basic cytolytic polypeptides (59–61 residue-long) that bind to cell membranes, causing a wide range of effects [16]. These include depolarization and necrosis of heart and skeletal muscles, lysis of blood and epithelial cells, and induction of toxicity in cortical neurons and various types of cancer cells [19]. They feature high homology in their amino acid sequences and their three-dimensional structure is characterized by high stability due to the presence of a network of hydrogen bonds, salt bridges and disulfide bonds [20,21]. Thus, CTX exhibit extreme thermal stability, resistance to pH-variation, and addition of denaturing agents and organic co-solvents [22]. All CTX exhibit antibacterial [21] and anticancer [23,24,25,26] activities. However, it is difficult to establish structure–activity relationships for them, because often even a single amino acid substitution within a CTX molecule may result in a noticeable change in activity [23]. Moreover, different conformational forms of a single CTX exhibit strikingly different membrane interactions [27]. From NMR studies, it is known that in an aqueous solution CTX with a single Pro residue in the N-terminal loop (loop-I) exhibit conformational equilibrium (Figure 1a) between major and minor forms [28,29,30]. The former is characterized by a trans-configuration in the X-Pro peptide bond (Figure 1a, left). The minor one features a cis-configuration in this bond (Figure 1a, right) and does not interact with detergent micelles and lipid membranes [30,31]. Recent molecular dynamics studies showed that the interaction between CTX possessing a single Pro residue within loop-I and zwitterionic lipid bilayers occurs by sequential embedding of loops I, II, and then III [32]. This means that the conformational configuration of loop-I is crucially important for the interaction of CTX with lipid membranes.

If CTX contain a pair of Pro residues in the first loop (Figure 1c), there is only one form in an aqueous solution (Figure 1b) [33]. These toxins were classified as group-I CTX [34] and are characterized by a banana-twisted shape of loop-I (Figure 1b). In part, this is due to the cis-configuration of the Pro8-Pro9 peptide bond (Figure 1c). Taking the above considerations into account, according to which a form with a cis peptide bond should lose membrane activity, we may suppose that CTX with a pair of prolines in loop-I should also exhibit lower membrane activity. The goal of this work is to verify this hypothesis.

In this work, we have isolated seven CTX from cobra *N. naja* and *N. haje* venoms. We identified their amino acid sequences and reconstructed their 3D-spatial structures, using homology modeling. Then, we determined the activity of all these toxins against either Gram-positive (*B. subtilis*) or Gram-negative bacteria (*E. coli*), as well as against a cancer cell line (human lung adenocarcinoma, A549). In addition, the capability of isolated CTX to induce calcein leakage from phospholipid liposomes formed of an equimolar mixture of dioleoylphosphatidylcholine (DOPC) and dioleoylphosphatidylglycerol (DOPG) was evaluated. We found that CTX with a Pro-Pro peptide bond in loop-I indeed exhibit attenuated membrane-perturbing activity in model membranes and lower cytotoxic activity compared to a single Pro residue CTX in this loop.

## 2. Results

**Figure 2:**
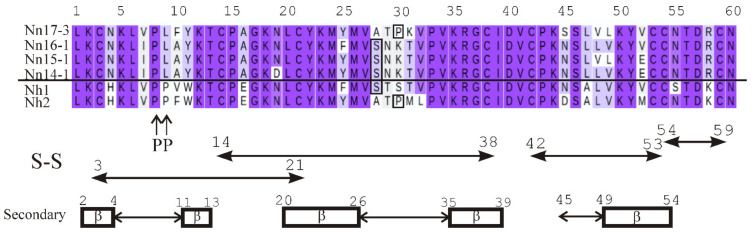
Amino acid sequences of the isolated toxins and elements of their secondary structure. The positions of disulfide bonds (S-S), beta-strands (rectangles with β inside) and extremities of the loops (double-headed arrows between rectangles) are indicated under the amino acid sequences. The numbers above the arrows correspond to the numbers of the first and the last amino acid residues. The numbering of the amino acid residues for all sequences is shown above. The residue Ser-28 and Pro-30, according to the presence of which CTX are classified as S- and P-type, respectively, are enclosed into boxes. The *N. haje* toxins with two prolines (indicated below the amino acid sequences) in loop-I are separated by the horizontal line from *N. naja* toxins with a single Pro residue in this loop. Similarity between the amino acid residues of the toxins is shown by color.

To isolate toxins, a three-step chromatographic procedure was used. Gel filtration on Sephadex G50 was the first step (Figure S1A). The so-called main toxic fraction V containing TFT was further separated by ion exchange chromatography on HEMA BIO 1000 CM column (Figure S1B) and fractions obtained were analyzed by mass spectrometry [35]. Fractions 14–18 were further purified by reversed phase HPLC. As a result, 5 toxins (Nn17–3, Nn18–3, Nn16–1, Nn15–1, Nn14–1) were purified (Table 1). In general, the separation of the main toxic fraction V (Figure S1A) by cation-exchange chromatography (Figure S1B) resembles the separation of the crude *N. naja* venom described in [36]. However, as the conditions of separation were different and the CTX possessed very close physical characteristics (charge, hydrophobicity etc.), the order for the elution of toxins might be very different and this is indeed what we observed here. After an additional step of toxin purification by reversed-phase HPLC in this work, their molecular masses were determined by mass spectrometry (Table 1). Determined masses of 4 toxins within errors corresponded to those of toxins described in [36]. The amino acid sequences of the toxins are given in Figure 2. The amino acid sequence of Nn16-1 cannot be identified using the known molecular masses, because alternative variants with identical molecular mass exist in the database (https://www.uniprot.org/ accessed on 15 December 2022). These are cytotoxin 3 (Uniprot code P24780) with a Val48-Leu49 pair and cytotoxin 2 *N. kaouthia* (Uniprot code P01445) with Leu 48-Val 49. X-ray analysis of the crystal structure of the toxin, namely its 2Fo-Fc electron density map at 2σ level for 47–52 region, allowed us to conclude that the correct order of the residues in this fragment is Leu48-Val49 [37]. Thus, the toxin is identical to cytotoxin 2 from *N. kaouthia* venom. The spatial structure of this toxin was solved recently by NMR spectroscopy in aqueous solution [38]. The determined mass of the toxin Nn18-3 did not match the mass of any known *N. naja* CTX or one from other snakes. However, taking into account the molecular weight of the toxin, its very basic nature and high activity similar to P-type CTX (see below), as well as the general characteristics of CTX amino acid sequences, we hypothesize its amino acid sequence (Table 1, footnote 1). Interestingly, in this work the order of the elution from the cation-exchange column for the CTX correlates with their isoelectric point (Table 1).

A pair of toxins was purified from *N. haje* venom (Nh1, Nh2 in Table 1) using a similar procedure. The gel-filtration profile for crude *N. haje* venom was similar to that obtained by Weise et al. [40]. After separation of the main toxic fraction (S3 in [40]) by cation-exchange chromatography, two prevailing fractions, similar to fractions V^II^1 and V^II^2 in [40] and called Nh1 and Nh2, were obtained. They were further purified by reversed-phase HPLC. The molecular masses of the toxins obtained in our work were equal to 6688.05 and 6850.33 Da, which correspond to the molecular masses of the toxins V^II^1 (P01455)—6688.11 Da and V^II^2 (P01462)—6850.38 Da.

The amino acid sequences of the isolated toxins, when aligned with CTX whose spatial structure is known, allow identification of the elements of their secondary structure (Figure 2, Table S1).

**Figure 3:**
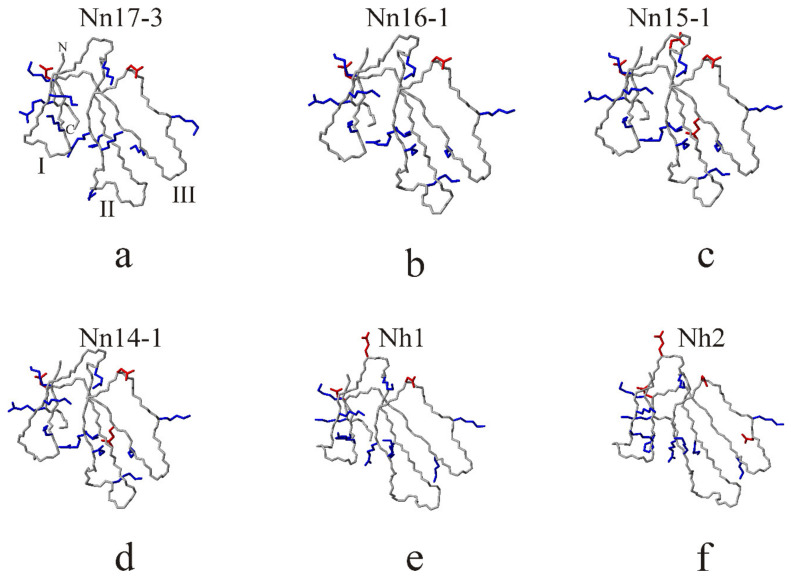
Spatial structures of the *N. naja* and *N. haje* CTX. In structures of *N. naja* toxins: 17–3 (**a**), 16–1 (**b**), 14–1 (**c**), and 15–1 (**d**), as well as *N. haje* toxins Nh1 (**e**)and Nh2 (**f**), only backbone and side-chains (heavy atoms) of the charged (positively charged Arg, Lys–blue, negatively charged Asp, Glu –red) amino acid residues are shown. In panel (**a**), the loops are numbered with Roman numerals. The orientation of CTX molecules in all the panels is identical, so the loop numbering is the same as in panel (**a**). In addition, C and N-termini of the molecules are marked. The backbones of the molecules are colored grey in all the panels. Note that the *N. naja* toxins and *N. haje* toxins are different primarily in the organization of loop-I (see Figure 1 for details).

CTX are highly homologous proteins and their amino acid sequences differ only by a few residues (Figure 2). Thus, their structural homology is expected, and homology modeling is the method of choice in predicting the spatial structure of CTX (Figure 3). The structures of CTX from *N. naja* venom (Figure 3a–d) exhibit close similarity. The CTX from *N. haje* venom (Figure 3e,f) differ from *N. naja* toxin due to the organization of loop-I (see Figure 1 for details). All CTX feature an unequal number of positive and negative residues (Figure 3).

**Figure 4:**
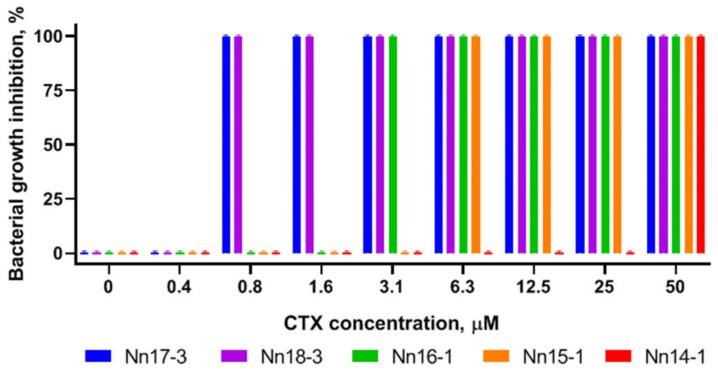
Response of *B. subtilis* to increasing concentrations of CTX from *N. naja* venom. For each concentration, a set of 5 bars of distinct color is shown. The experiments for CTX Nh1, Nh2 from *N. haje* venom, featuring a Pro-Pro bond in loop-I, did not reveal any activity in the studied concentration range.

Further, antibacterial and cytotoxic activities of isolated CTX were determined. CTX from *N. naja* do not exhibit antibacterial activity against Gram-negative bacteria *E. coli* and Gram-positive bacteria *S. aureus* at concentrations up to 50 μM. At the same time, they possess bactericidal activity (minimum inhibitory concentration (MIC) = minimum bactericidal concentration (MBC)) against Gram-positive *B. subtilis* in the micromolar concentration range (Table 1). The concentration-dependent inhibition of *B. subtilis* by CTX is shown in Figure 4.

**Figure 5:**
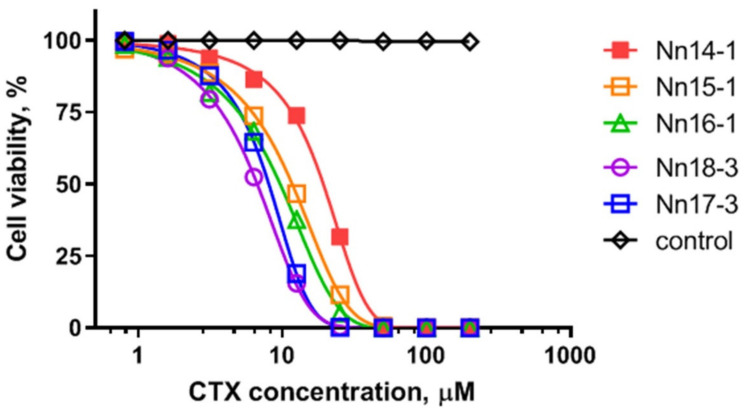
Dependence of A549 cell survival on the concentrations of CTX from *N. naja* venom after 3 h of incubation. The black curve corresponds to the control experiment, where no CTX was added.

In addition, all CTX showed cytotoxic activity against human lung adenocarcinoma A549 (Table 1). The concentrations of toxins resulting in the death of 50% of cells (LD_50_) spanned the low micromolar range (~5–17 μM) (Table 1). The dependence of A549 cell survival on the concentrations of the *N. naja* CTX is illustrated in Figure 5. The cytotoxic activities of CTX Nh1 and Nh2 from *N. haje* venom was studied in the work of Feofanov et al. [23].

**Figure 6:**
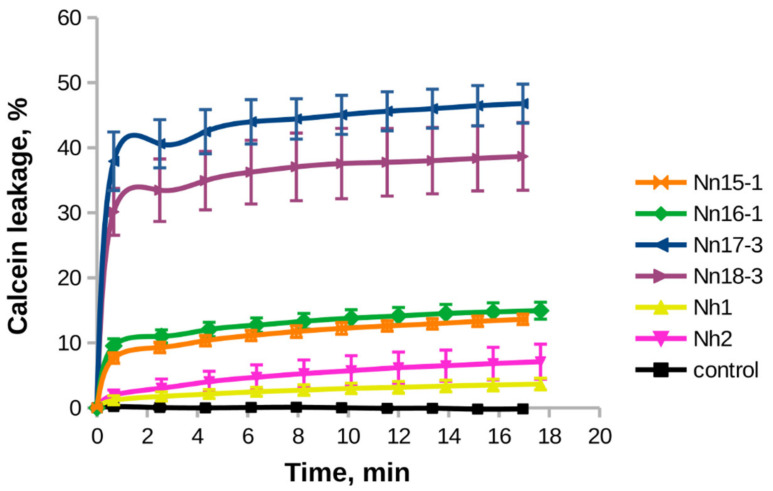
Time-dependence of calcein leakage from DOPC/DOPG (1:1) unilamellar liposomes induced by CTX. The concentration of the total lipid was 0.1 mM. The concentration of added CTX was 1 µM. The vertical bars represent an experimental error, estimated by averaging after three measurements. The black curve corresponds to the control experiment, where no CTX was added to the liposomes.

The membrane-perturbing activity of CTX was investigated in model membranes, composed of an equimolar mixture of anionic and zwitterionic phospholipids, DOPG and DOPC. The studied toxins (except for Nn14-1, isolated in a small amount) induced calcein leakage from unilamellar liposomes in a time-dependent manner (Figure 6). The increasing amount of added toxin resulted in an increase in the leakage (Figure S2). In the range of the investigated lipid-to-peptide ratio (400:1–50:1), the calcein leakage was maximal for toxins Nn18-3 and Nn17-3 and minimal for Nh1 and Nh2 (Figure 6). The toxins Nn16-1 and Nn15-1 gave medium values of calcein leakage (Figure 6).

## 3. Discussion

According to a recent study, the proteome of *N. naja* contains 19 TFT, of which 10 are CTX and cardiotoxin-like basic proteins (CLBP) [41]. However, a border between CTX and CLBP, a separate group of TFT, is not well defined [42]. To discern between CTX and CLBP, we suggest the following definition for CTX (assuming cytotoxins = cardiotoxins): these are 59–61 residue-long three-finger folded toxins with eight cysteine residues and at least one methionine residue. From this viewpoint, only seven CTX are presented in Figure 5 of the above-cited work [41], while three others are 61–62 residue-long CLBP. In the present work, we identified five CTX from *N. naja* venom (Table 1). Only two of them, namely the toxins Nn17-3 and Nn16-1, are listed in Figure 5 of the work of Suryamohan et al. [41]. In addition, we investigated a pair of CTX from *N. haje* venom (Table 1). The cytotoxic activity of these toxins was investigated earlier [23].

**Figure 7:**
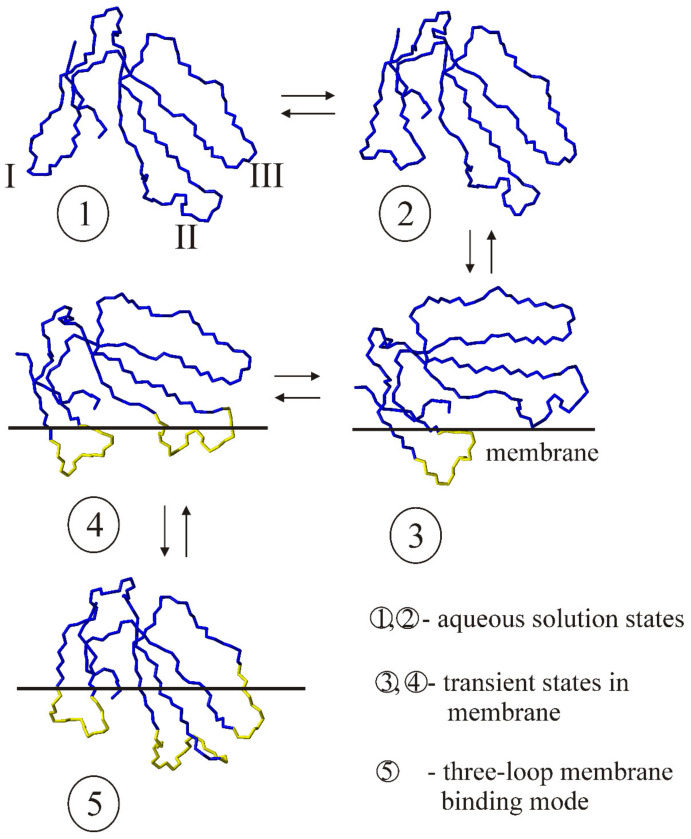
Conformational equilibrium of CTX with a single Pro residue in loop-I in aqueous solution and their binding to a lipid membrane. The numbers in circles below the models correspond to the structural states of cytotoxin 1 from *N. oxiana* (CT1No) in an aqueous solution and lipid membrane. The structures were determined by NMR spectroscopy in an aqueous solution and detergent micelles. Only backbone is shown for all the structural models. The pdb code of state **1** determined for the recombinant CT1No is 5LUE (Met0-residue was removed). The bond Val7-Pro8 in state **1** is in cis-configuration, and trans-configuration in all other models (**2**–**5**) (see also inserts in Figure 1a, for the details of the conformation of this bond). The loops are marked with Roman numerals for state **1**. The numbering of the loops is from left to right for all other models. States **2** and **5** are models 5NPN and 5NQ4, respectively. The membrane-interacting loops of the models, corresponding to states **3**–**5**, are marked in yellow. The membrane surface is schematically shown with a horizontal line. The transition from state **3** to **4** is accompanied with conformational changes within loop-II of the molecule.

As discussed above, CTX feature a three-finger folded spatial structure (Figure 1, Figure 2 and Figure 3). According to NMR data, conformational equilibrium between the two forms is observed in an aqueous solution [28,29]. This arises due to cis-trans isomerism of the X-Pro8 peptide bond in loop-I of the molecule (Figure 1a, Figure 7, panels 1, 2).

Only a conformer with a trans-configuration of this bond is capable of partitioning into the lipid membrane [30,31] (Figure 7, panel 3). CTX partitioning into lipid membranes occurs strictly in the following order: first, loop-I embeds in lipid membrane (Figure 5, panel 3). Then, loop-II (Figure 7, panel 4) and loop-III (Figure 7, panel 5) join it, consecutively. This result was obtained using a molecular dynamics simulation study of the incorporation of cytotoxin 2 from *N. oxiana* in a palmitoyloleoylphosphatidylcholine membrane [32]. Importantly, for S-type CTX, embedding of loop-II in the membrane is accompanied with conformational change within the fragment surrounding Ser28-residue (Figure 7, transition from panel 3 to 4). This is why this residue is predetermining the S-type of CTX. For P-type CTX lacking this residue, the corresponding transition occurs without conformational change within this fragment [31]. Therefore, for S-type CTX, the states with loop-I embedded into the membrane (Figure 7, panel 3) will be statistically more populated than the states with two or three embedded loops (Figure 7, panels 4, 5). There is no such detailed information for CTX of group-I bearing a pair of Pro residues in loop-I. Toxin γ from *N. nigricollis* containing two neighboring prolines in loop-I was shown to interact with dodecylphosphocholine micelle via all three loops [33]. However, in the micelle-bound state, two sets of cross peaks are seen in the NMR spectra. This likely indicates a slow in the NMR time-scale conformational equilibrium between the cis peptide Pro8-Pro9 bond and its trans form. For CTX with a single Pro residue in loop-I, such an equilibrium is observed in aqueous solution [28]. For group-I CTX, a transition from state 1 to 3 occurs (Figure 7) because there is no minor form in aqueous solution [33]. Thus, state 1 is more populated than state 3 for group-I CTX, compared to those with a single Pro residue within this loop. This means that the membrane-perturbing activity of group-I CTX should be lower than their counterparts with a single Pro residue.

The antibacterial activity of CTX (Table 1) agrees qualitatively with our previous findings [39]. CTX killed neither *E. coli* nor *S. aureus* cells. Only Gram-positive bacteria, such as *B. subtilis* (this work) and *M. luteus* (previous work [39]), are influenced. Both P-type toxins Nn17-3 and Nn18-3 feature similar antibacterial activity (Table 1). The S-type CTX, Nn14-1, Nn15-1, and Nn16-1, manifest lower activity. Among them, the toxin Nn14-1 demonstrates the lowest activity, likely due to its low net electrical charge [39]. Charge distribution over molecule surface was shown to be important for this activity, as CLBP showed a high effect due to optimal positioning of positively charged residues [43]. Compared to *N.naja* CTX, the toxins Nh1 and Nh2 from *N. haje* venom with two prolines in loop-I show the lowest antibacterial activity, although their charges are identical to those of Nn15-1 and Nn14-1, respectively (Table 1).

For a number of CTX, toxic activity against human adenocarcinoma A549 cells was investigated [23]. All CTX were found to be located in the lysosomes but not on the plasma membrane of the cells. In this work, the maximal activities were observed for Nn18-3 and Nn16-1 toxins (Table 1). The toxins Nn17-3 and Nn15-1 manifest a slightly lower activity. The lowest activity from *N. naja* toxins is exhibited by Nn14-1. Again, both Nh1 and Nh2 exhibit the lowest cytotoxic effect, and their LD_50_ values are two orders of magnitude higher than those for *N. naja* toxins (Table 1).

Six of seven isolated CTXs were investigated in model membranes. They induced leakage of calcein from calcein-loaded unilamellar vesicles, composed of an equimolar mixture of DOPC and DOPG (Figure 6). CTX, according to their leakage activity, are positioned in the following order: Nn18-3 ≈ Nn17-3 > Nn16-1 ≈ Nn15-1 > Nh2 ≈ Nh1. Again, the lowest activity was exhibited by CTX Nh2 and Nh1, both featuring the Pro-Pro bond in loop-I. Interestingly, a similar order of activity was observed in antibacterial tests. A slightly modified row in cytotoxic activities against A549 cells is likely due to the unequal capacity of different CTX to be captured inside lysosomes. This effect depends on the specific distribution of positively charged and hydrophobic domains on the surface of the toxin molecules [44].

Taking into account the antibacterial and cytotoxic activities (Table 1), as well as the ability of CTX to cause leakage in model membranes (Figure 6), we may conclude that the low activity of CTX Nh1 and Nh2 is due to their lower capacity for binding to lipid bilayers (Figure 7 and discussion above).

## 4. Conclusions

In the present work we have isolated five CTX from cobra *N. naja* and two from *N. haje* venoms. We have identified their amino acid sequences and determined the activity of all these toxins against either Gram-positive (*B. subtilis*) or Gram-negative bacteria (*E. coli*), as well as against cancer cells (human lung adenocarcinoma, A549). We also evaluated their capability to induce calcein leakage from phospholipid liposomes, formed of an equimolar mixture of DOPC and DOPG. We found that CTX with a Pro-Pro peptide bond in loop-I, including both CTX from *N. haje* venom, exhibit attenuated membrane-perturbing activity in model membranes, and lower cytotoxic and antibacterial activities, compared to CTX from *N. naja* venom containing a single Pro residue in loop-I. This may indicate that both antibacterial and cytotoxic activities of CTX arise due to their interaction with the plasma membrane of bacterial cells, or cellular organelles of the cancer cells. The absence of antibacterial activity in CTX against Gram-negative bacteria and some Gram-positive ones is likely explained by incapability of CTX to reach their plasma membrane due to unfavorable interactions with the outer membrane of the bacteria, or their peptidoglycan layer.

## 5. Material and Methods

### 5.1. Chemicals

Cobra venoms were obtained from snakes kept in captivity at 25 ± 26 °C and fed mainly with mice and rats. The snakes were milked by manual gland massage, and the venom obtained in this way was dried over anhydrous CaCl_2_ and stored at –20 °C. All procedures with snakes were approved by the Committee for the Care and Use of Laboratory Animals of the Shemyakin-Ovchinnikov Institute of Bioorganic Chemistry of the Russian Academy of Sciences. Protocol-application number 324/2021 dated 23 June 2021. The A549 cell line was obtained from the D.I. Ivanovsky Institute of Virology of the Russian Academy of Medical Sciences (Moscow, Russia). Acetonitrile was purchased from Catrosa Reaktiv LLC (Moscow, Russia), and trifluoroacetic acid from Merck KGaA (Darmstadt, Germany). Calcein sodium salt was a product of Serva (Heidelberg, Germany). Other salts were of analytical grade or higher; they were obtained from local suppliers.

### 5.2. Isolation of Cytotoxins

A 600 mg sample of dried *N. naja* venom was dissolved in 0.1 M ammonium acetate buffer, pH 6.2, and applied to a Sephadex G50s column (4.5 × 150 cm) equilibrated in the same buffer. The column was eluted at flow rate 32 mL/min. The fractions obtained were pooled as shown in Figure S1A. Fraction V was further separated on a HEMA BIO 1000 CM column (8 × 250 mm) (Tessek, Prague, Czech Republic) in an ammonium acetate gradient from 5 to 700 mM (pH 7.5) in 140 min at flow rate 1.0 mL/min (Figure S1B). Fractions 14–18 were freeze-dried and further purified by reversed-phase chromatography on a Jupiter C18 column (10 × 250 mm, Phenomenex, Torrance, CA, USA) in a gradient of acetonitrile 25–35% in 60 min in the presence of 0.1% trifluoroacetic acid, at a flow rate of 2.0 mL/min. After freeze-drying, the obtained proteins were used for further studies. Molecular masses of the isolated products were determined by mass spectrometry [35]. A similar procedure was used to isolate cytotoxins from *N. haje* venom.

### 5.3. Antibacterial Activity

The study of the antibacterial activity of CTX was conducted by the two-fold microtiter broth dilution assay in 96-well sterile plates at a final volume of 100 μL. Bacteria (*Bacillus subtilis VKM B-501*, *Escherichia coli C600*, and *Staphylococcus aureus 209-P* strains) were cultured overnight in the MH (Mueller Hinton Broth, Sigma) medium at 37 °C. Mid-log phase cultures were diluted to 5×10^3^ colony-forming units/mL. Toxins in the 0.4–50 µM concentration range were added to the suspension of bacterial cells and incubated for 20 h (37 °C, 100% humidity, gentle mixing). Growth inhibition was determined by measuring absorbance at 595 nm. Minimal inhibitory concentrations (MICs) were determined as the lowest concentrations of peptide that caused total inhibition of bacteria growth. The toxins, a control (bacteria without peptides), and a sterility control (MH medium) were tested in triplicate.

### 5.4. Cytotoxic Activity

Human lung adenocarcinoma A549 cells were cultured in DMEM (Dulbecco’s modified Eagle medium) medium containing 2 mM L-glutamine and 8% fetal calf serum (so-called complete medium) at 100% humidified 5% CO_2_ atmosphere at 37 °C. Reseeding was performed two times a week. On the day before the experiment, the cells were seeded in 96-well flat-bottom plates (15,000 cells per well). Cytotoxicity was assessed after incubation of the cells with toxins by varying their concentration in the range of 0.8–200 µM with an incubation time of 3 h. All experiments were repeated three times. The percentage of surviving cells was assessed by fluorescence microscopy, as described elsewhere [23]. Control cells were incubated with an appropriate amount of water for 3 h.

### 5.5. Structure Modeling of the Toxins

The spatial structures of all CTX was obtained via homology modeling using SWISS modeling workspace [45]. The templates were chosen manually, taking into consideration the amino acid sequence identity with the homologues whose spatial structure had been determined either by NMR spectroscopy or by X-ray crystallography. The information about the templates is summarized in Table S1.

### 5.6. Calcein-Loaded Liposome Preparation

To control lipid bilayer integrity on incubations with different toxins, liposome samples encapsulating calcein at the self-quenching concentration were prepared. Liposomes composed of an equimolar mixture of DOPC/DOPG (Lipoid, Germany) were prepared by standard extrusion method. Briefly, dry lipid films were hydrated in PBS (1.5 mM KH_2_PO_4_, 1.1 mM NaH_2_PO_4_, 6.3 mM Na_2_HPO_4_, 2.7 mM KCl, and 136.8 mM NaCl; pH 7.0) with 80 mM calcein and subjected to 6–10 cycles of freezing/thawing (liquid nitrogen/+40 °C). The suspension was then extruded at ambient temperature through two stacked polycarbonate membrane filters with pore sizes of 100 nm (Nucleopore), 10 times, on a Mini-Extruder (Avanti Polar Lipids). After extrusion, non-encapsulated calcein was removed by size exclusion chromatography on a Sephadex G-50 column equilibrated in PBS. To control the final liposome concentration, calcein absorbance peaks were registered upon liposome disruption with at least a 20-fold volume of ethanol (calcein: λ_max_ = 497 nm, ε~74,000 M^−1^ cm^−1^) on an SF-2000 spectrophotometer (OKB Spectr, Russia). Before measuring, the liposomes were diluted to adjust the absorbance of the calcein to less than 0.05 optical density to avoid the inner filter effect. The final concentration of total liposome lipids was in the range of 10^−4^–10^−5^ M. The size of the liposomes after chromatography was controlled by dynamic light scattering using a Litesizer 90Plus particle analyzer (Anton Paar, Austria) in at least three runs per sample. Mean liposome diameter was in the range of 155–160 nm. Formulations were stored at 4 °C and used for the tests within 10 days. Leakage of calcein from the liposomes and its dilution results in the dequenching of the fluorophore and an increase in the fluorescence signal. The percentage of calcein released (CR, %) was calculated according to the equation:

CR = (I_i_/I_T_ − I_0_/I_T_)/ 1 − I_0_/I_T_) × 100%,

where I_i_ is the fluorescence intensity at a given time point, I_0_ is the intensity of the untreated liposomes, and I_T_ is the totally dequenched calcein fluorescence after the addition of Triton X-100 (10 μL of 20% Triton X-100 was added to each sample of 200 μL). The measurements were performed on a GloMaxR-Multi instrument (Promega, Madison, WI, USA ) using the blue fluorescence optical kit (λ_ex_ = 490 nm, λ_em_ = 510–570 nm) in 96-well plates.

## Figures and Tables

**Table 1:** Physico-chemical properties of CTXs and their antibacterial and cytotoxic activities.

Toxin Name	Uniprot Codes of the Identified Toxins	Molecular Weight, Da ^1^	Isoelectric Point	Electrical Charge	P/S-Type	Antibacterial (*B. subtilis*) Effect, MIC, µM	Cytotoxic Effect (Against A549 cells), LD_50_, µM
Nn17-3	P01440	6755.2	9.36	9	P	0.8	7.4 ± 0.1
Nn18-3	-	6782.5	9.36	9 ^2^	P ^2^	0.8	5.0 ± 0.5
Nn16-1	P01445	6736.5	9.38	9	S	3.1	5.6 ± 1.5
Nn15-1	P01447-1	6783.5	9.24	8	S	6.3	7.9 ± 0.4
Nn14-1	P86382-1	6783.7	9.11	7	S	50	17.4 ± 0.8
Nh1 ^3^	P01455	6688.1	9.15	8	S	>50 (80 ^4^)	132 ± 9 ^5^
Nh2 ^3^	P01462	6850.3	8.99	7	P	>50 (40 ^4^)	116 ± 6 ^5^

^1^ Experimental error is ±0.2 for all the values; ^2^ These features refer to the hypothesized amino acid sequence of this toxin: LKCNKLVPLFYKTCPKGKNLCYKMYMVAAPTVPVKRGCINVCPKNSLVLKYECCNTNKCN; ^3^ These toxins feature double Pro-bond in the loop-I; ^4^ Activity against *M. luteus* [39]; ^5^ According to the data in [23].

## Data Availability

All data obtained in this study are contained within the article.

## Supplementary Materials

The following supporting information can be downloaded at: www.mdpi.com/article/10.3390/toxins15010006/s1, Table S1: Toxins and their structural homologues, used as templates for building 3D-models, Figure S1: Isolation of *N. naja* cytotoxins, Figure S2: Time-dependence of leakage from calcein loaded DOPC/DOPG (1:1) liposomes, induced by addition of CTX, studied in the current work.

## Abbreviations

CLBP, cardiotoxin-like basic protein(s); CT1No, cytotoxin 1 from *N. oxiana*; CTX, cobra cardiotoxin(s) (cytotoxin(s)); DOPC, dioleoylphosphatidylcholine; DOPG, dioleoylphosphatidylglycerol; MBC, the minimum bactericidal concentration (the lowest concentration of CTX causing the death of all bacteria); MIC, the minimum inhibitory concentration (the lowest concentration of CTX completely inhibiting the reproduction of bacteria); PDI, polydispersity index; TFT, three-finger toxin(s).
