# Supplementary material for: Membrane-Disrupting Activity of Cobra Cytotoxins Is Determined by Configuration of the N-Terminal Loop
Source: Toxins (Basel). 2022 Dec 20;15(1):6. doi: 10.3390/toxins15010006 (PMC9866941; doi:10.3390/toxins15010006)
Supplement: Supplementary file 1 [file toxins-15-00006-s001.zip › toxins-2080489-supplementary-done.docx]

Supplementary Materials: Membrane-Disrupting Activity of Cobra Cytotoxins Is Determined by Configuration of the
N-Terminal Loop

Peter V. Dubovskii, Anastasia A. Ignatova, Anna S. Alekseeva, Vladislav G. Starkov, Ivan A. Boldyrev,
Alexey V. Feofanov and Yuri N. Utkin

**Table S1.** Toxins and their structural homologues, used as templates for building 3D-models.

| Toxin | Name of the homologous toxin | Amino acid sequence of the homologous toxin^1^ | Sequence identity | PDB code of the homolog |
| --- | --- | --- | --- | --- |
| Nn17-3 | A3^2^ | LKCNKLVPLFYKTCPAGKNLCYKMFMVATPKVPVKRGCIDVCPKSSL**LV**KYVCCNTDRCN | 97% | 2BHI |
| Nn16-1 | CT2Nk^3^ | LKCNKLIPLAYKTCPAGKNLCYKMFMVSNKTVPVKRGCIDVCPKNSLVLKYVCCNTDRCN | 100% | 7O2K |
| Nn15-1 | CT2Nk^3^ | LKCNKLIPLAYKTCPAGKNLCYKM**F**MVSNKTVPVKRGCIDVCPKNSL**LV**KY**V**CCNTDRCN | 93% | 7O2K |
| Nn14-1 | CT2Nk^3^ | LKCNKLIPLAYKTCPAGK**N**LCYKM**F**MVSNKTVPVKRGCIDVCPKNSLLVKY**V**CCNTDRCN | 95% | 7O2K |
| Nh1 | Toxin-γ | LKC**NQ**L**I**PPFWKTCP**K**GKNLCYKM**T**M**R**A**A**PM**V**PVKRGCIDVCPK**S**S**L**L**I**KYMCCNTDKCN | 73% | 1TGX |
| Nh2 | Toxin-γ | LKC**NQ**L**I**PPFWKTCP**K**GKNLCYKM**T**M**R**A**A**PM**V**PVKRGCIDVCPK**S**S**L**L**I**KYMCCNTDKCN | 82% | 1TGX |

^1^- The amino acid residues different from those in the sequence of N. naja/haje toxin are shown in bold and underlined;

^2^- Cytotoxin 3, Taiwan (*N. atra*) cobra;

^3^- Cytotoxin 2 from *N. kaouthia* venom.


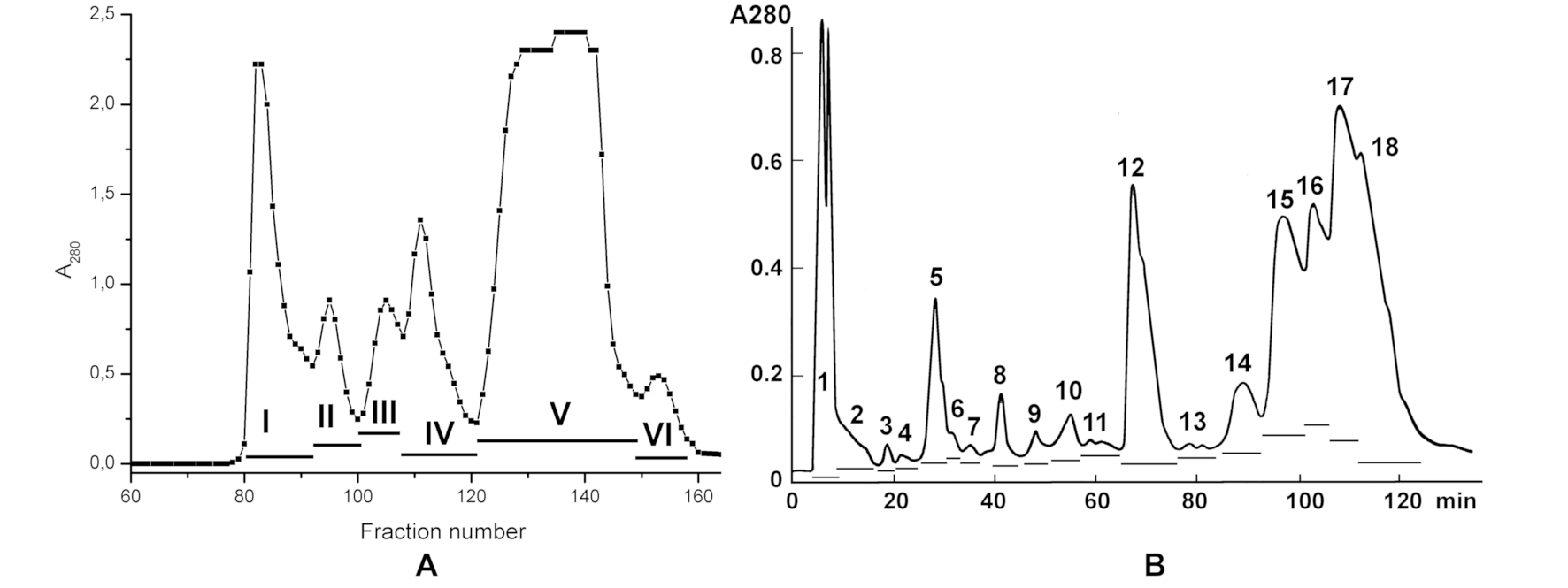


**Figure S1.** Isolation of *N. naja* cytotoxins. (A) Separation of the crude *N. naja* venom by gel-filtration on Sephadex G50 column. (B) Separation of fraction V by ion exchange chromatography on HEMA BIO 1000CM column.


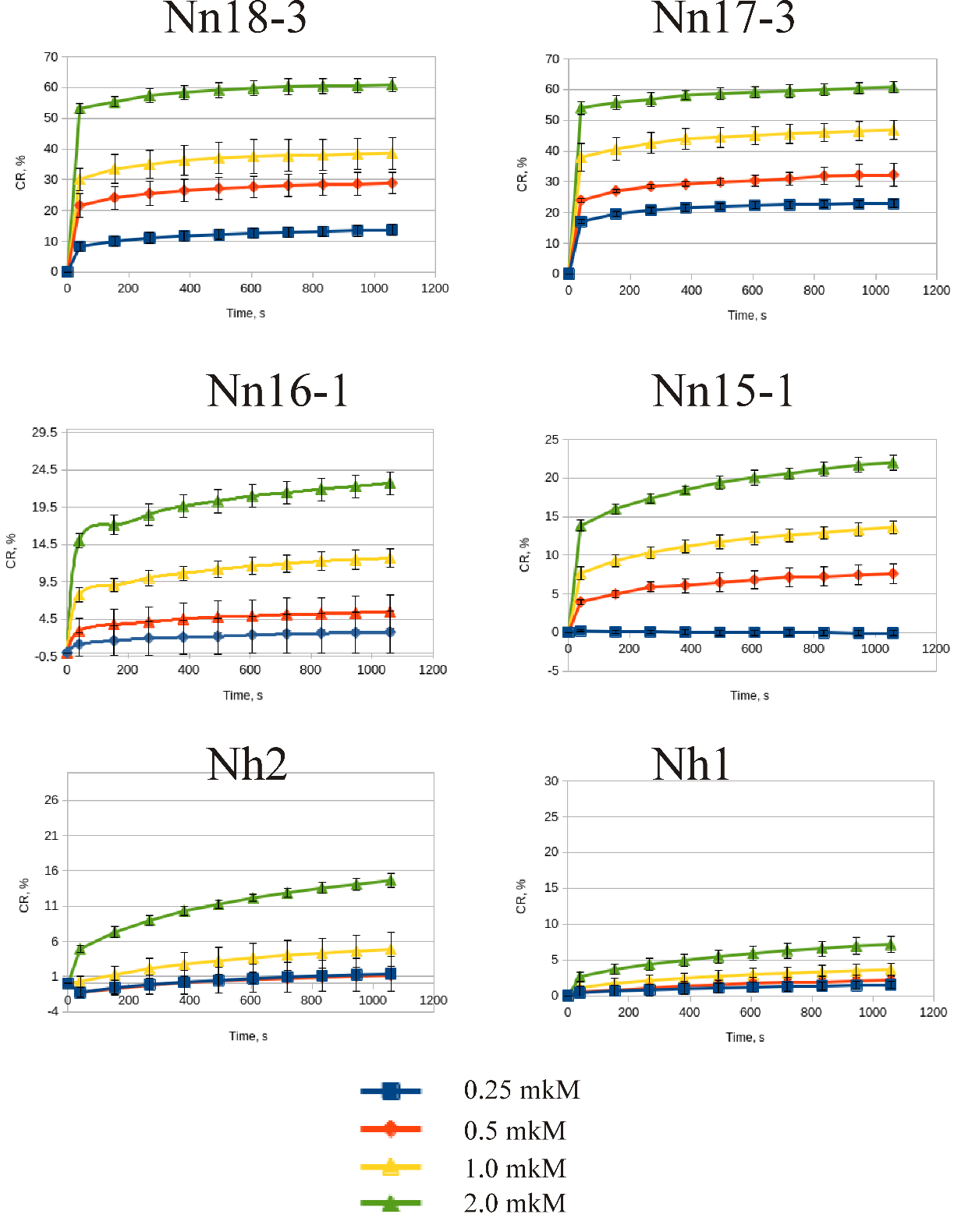


**Figure S2.** Time-dependence of leakage (CR, %, see Formula for its calculation in the Material and methods section) from calcein loaded DOPC/DOPG (1:1) liposomes, induced by addition of CTX, studied in the current work (see Fig. 2, Table 1 for their amino-acid sequences). Each panel corresponds to the toxin, indicated above the panel. The total concentration of lipid was 0.1 mM in all the experiments. The concentration of the added toxin is color-coded and is indicated below the panels.
